# Comparison of risk factors for squamous cell and adenocarcinomas of the cervix: a meta-analysis

**DOI:** 10.1038/sj.bjc.6601764

**Published:** 2004-04-13

**Authors:** A Berrington de González, S Sweetland, J Green

**Affiliations:** 1 Cancer Research UK Epidemiology Unit, Gibson Building, Radcliffe Infirmary, Woodstock Road, Oxford OX2 6HE, UK

**Keywords:** cervix neoplasms, risk factors, adenocarcinoma, squamous cell carcinoma, smoking

## Abstract

While most cancers of the uterine cervix are squamous cell carcinomas, the relative and absolute incidence of adenocarcinoma of the uterine cervix has risen in recent years. It is not clear to what extent risk factors identified for squamous cell carcinoma of the cervix are shared by cervical adenocarcinomas. We used data from six case–control studies to compare directly risk factors for cervical adenocarcinoma (910 cases) and squamous cell carcinoma (5649 cases) in a published data meta-analysis. The summary odds ratios and tests for differences between these summaries for the two histological types were estimated using empirically weighted least squares. A higher lifetime number of sexual partners, earlier age at first intercourse, higher parity and long duration of oral contraceptive use were risk factors for both histological types. Current smoking was associated with a significantly increased risk of squamous cell carcinoma, with a summary odds ratio of 1.47 (95% confidence interval: 1.15–1.88), but not of adenocarcinoma (summary odds ratio=0.82 (0.60–1.11); test for heterogeneity between squamous cell and adenocarcinoma for current smoking: *P*=0.001). The results of this meta-analysis of published data suggest that squamous cell and adenocarcinomas of the uterine cervix, while sharing many risk factors, may differ in relation to smoking. Further evidence is needed to confirm this in view of the limited data available.

Most cancers of the uterine cervix are squamous cell carcinomas, but the relative and absolute incidence of adenocarcinoma has risen in recent years and adenocarcinomas now account for about 20% of incident invasive cervical cancers in screened populations worldwide (Sasieni and Adams, 2001). It remains unclear to what extent risk factors identified for squamous cell carcinoma of the cervix are shared by cervical adenocarcinomas (Parazzini and La Vecchia, 1990; Kjaer and Brinton, 1993; Altekruse *et al*, 2003; Green *et al*, 2003). While infection with the human papillomavirus (HPV) appears to be the most important cause of both types of cervical cancer (Walboomers *et al*, 1999; Clifford *et al*, 2003), some controlled studies have found differences between adenocarcinoma and squamous cell carcinoma in the importance of other factors such as smoking (Lacey *et al*, 2001; Green *et al*, 2003) and reproductive factors (Altekruse *et al*, 2003). Individual studies have generally been limited by small numbers of adenocarcinoma cases and in some instances by lack of adjustment for confounding factors. In the 10 years since this subject was last reviewed (Parazzini and La Vecchia, 1990; Kjaer and Brinton, 1993), a number of new studies have been published. In this meta-analysis of published data, we have combined results from those controlled studies that provided a direct comparison between risk factors for squamous cell and adenocarcinoma, to assess the current evidence.

## MATERIALS AND METHODS

Studies were identified through searches of MEDLINE (1966–June 2003, using combinations of the search terms ‘cervix neoplasms’, ‘risk factors’, ‘adenocarcinoma’ and ‘squamous cell carcinoma’) and of bibliographies of identified papers. We included any controlled study that provided the age-adjusted odds ratios and 95% confidence intervals (CIs) for both adenocarcinoma (including adenosquamous carcinoma) and squamous cell carcinoma of the cervix (invasive or *in situ*) for at least one of the following risk factors (but not necessarily in the same publication): duration of oral contraceptive use, smoking, reproductive factors and sexual behaviour. Studies providing information on only one of the two histological types were not included, to ensure that any potential differences between the types were not due to study design or setting. No limit was placed on the number of cases. The most adjusted odds ratio available was used for analysis. In most studies, oral contraceptive use was not further defined and may include combined and progestagen-only oral contraceptives; however, the large majority of oral contraceptive users in these studies are likely to have used combined preparations (IARC, 1999).

### Statistical methods

The odds ratios from each study were grouped into the closest of the prespecified categories for each risk factor (e.g. for duration of oral contraceptive use <5, 5–9 and 10+ years). To enable the results for the studies that had been divided into more categories to be included, it was necessary to combine some of the categories using a method for combining nonindependent strata (Berrington and Cox, 2003).

The summary (odds ratios, OR) for the pooled data were calculated under a fixed effects model using the method of empirically weighted least squares, where the weights are defined as the inverse of the variance of the log odds ratios (Cox and Snell, 1989). Heterogeneity between individual study results and between summary risk estimates for the two histological types was also calculated using this method.

In Figure 1

**Figure 1:**
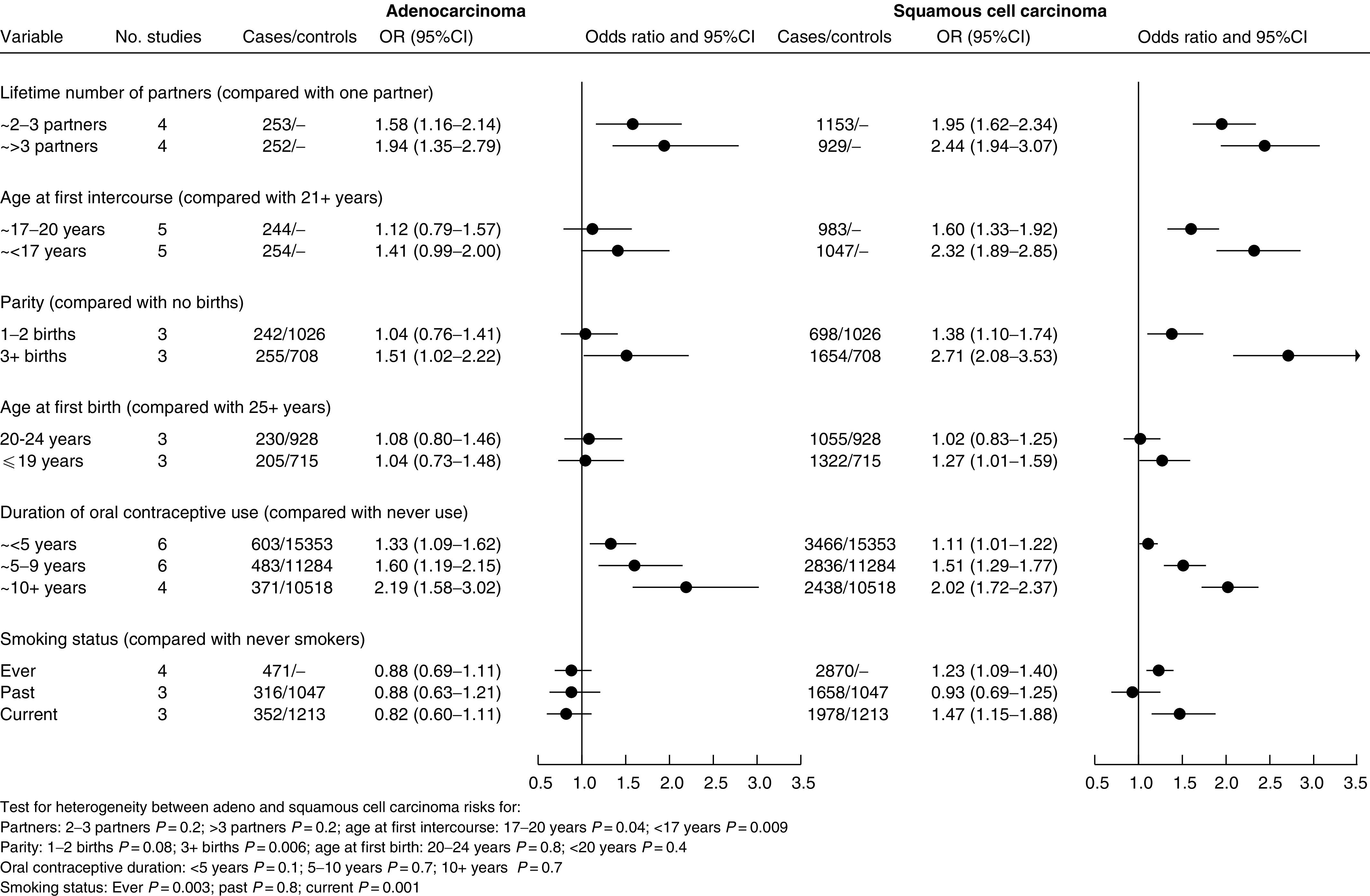
Summary ORs and 95% CIs for cervical cancer in relation to sexual behaviour, reproductive factors, oral contraceptive use and smoking status.

, summary OR for groups of studies are shown as black circles whose size does not represent the amount of data available. In Figure 2

**Figure 2:**
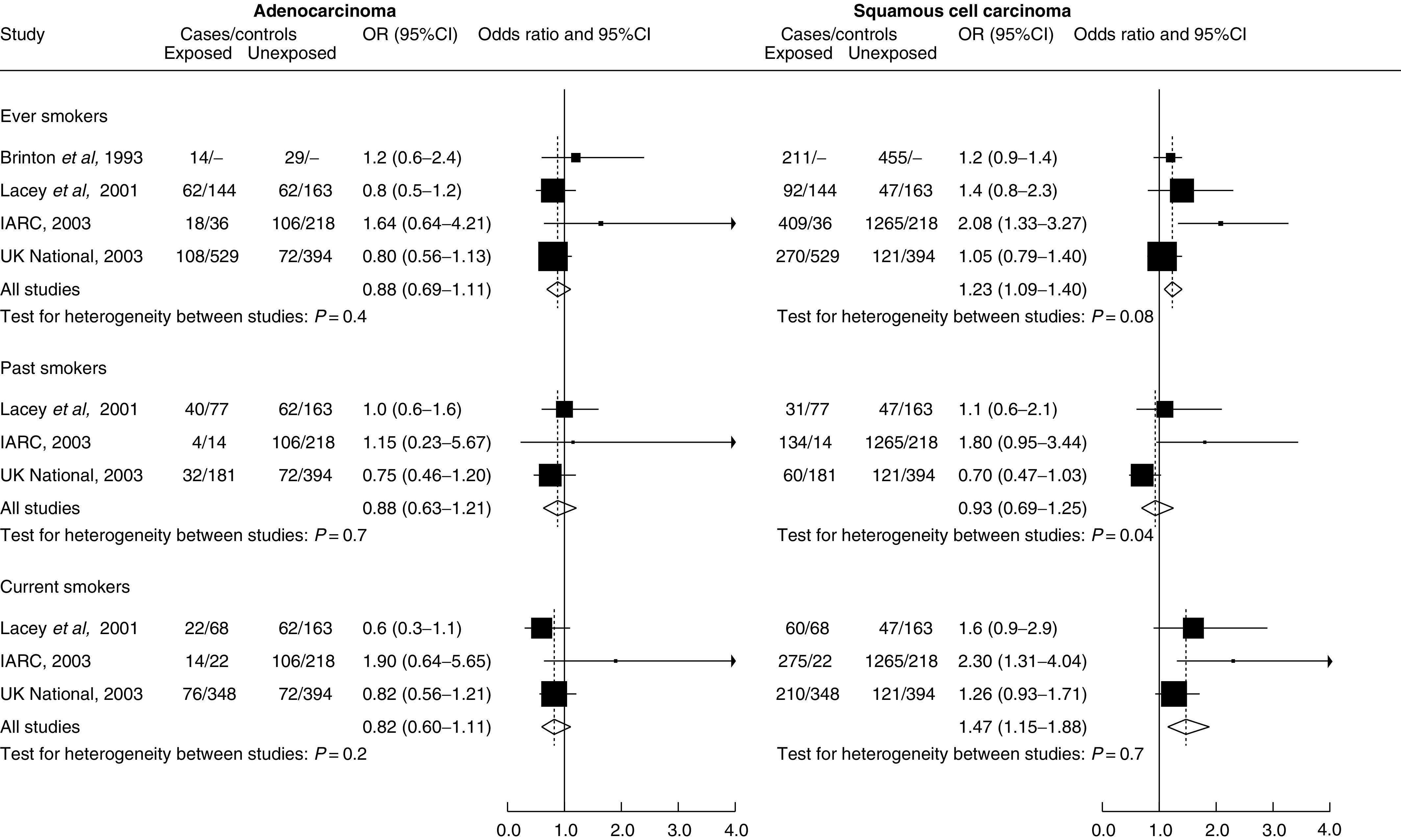
Odds ratios and 95%CIs for cervical cancer for ever, past and current smokers *vs* never smokers.

, OR for individual studies are plotted as black squares whose size is inversely proportional to the variance of the logarithm of the odds ratios diamonds represent the summary odds ratios with 95% CIs indicated by their horizontal extent.

## RESULTS

Data were available from six case–control studies: by Brinton and co-workers in the USA (Brinton *et al*, 1986) and Latin America (Brinton *et al*, 1990, 1993); the World Health Organisation (WHO) multicentre study (WHO, 1985; Thomas and Ray, 1996); a multicentre study by Lacey and co-workers in the USA (Lacey *et al*, 1999, 2001; Altekruse *et al*, 2003); a pooled analysis from the International Agency for Research on Cancer (IARC) (Munoz *et al*, 2002; Plummer *et al*, 2003) of data from 10 individual studies, of which two (Chichareon *et al*, 1998; Ngelangel *et al*, 1998) were included individually in analyses for which the pooled IARC data were not available; and the UK National Case–Control Study of Cervical Cancer (Green *et al*, 2003). In total, data were available for 5649 cases of squamous cell carcinoma, 910 cases of adenocarcinoma and 17 384 controls. Details of the studies are given in Table 1

**Table 1:** Studies included in published data meta-analysis of risk factors for squamous cell and adenocarcinoma of the cervix

**Study**	**Date of diagnosis**	**Histology**	**Cases squamous**	**Adeno**	**Controls**	Results adjusted for*									**Included in meta-analysis of**
						**SP**	**AFI**	**Py**	**HPV**	**OC**	**Sm**	**SES**	**Eth**	**Ctr**	
Brinton *et al* (1986), USA	1982–1984	Invasive	417	62	789	No	Yes	No	No	No	No	No	Yes	NA	OC use
Brinton *et al* (1990/1993), Latin America	1986–1987	Invasive	667	61	1413	Yes	Yes	No	No	No	No	No	No	No**	SmokingOC use partners/AFI
WHO (1993/1996), multicentre	1979–1988	Invasive	2361	377	13 644	No	No	Yes	No	No	No	No	No	Yes#	OC use
Lacey *et al* (1999–2003), USA	1992–1996	Invasive/*in situ*	91 inv 48 *in situ*	91 inv 33 *in situ*	307	Yes	No	Yes	Yes	No	No	Yes	Yes	NA	All
IARC pooled (2002/2003^+^), multicentre	1985–1997	Invasive/*in situ*	1463 inv 211 *in situ*	124 inv	254	Yes	Yes	Yes	Yes	Yes	Yes	Yes	No	Yes	Smokingparity/AFB
							
Chichareon *et al* (1998), Thailand	1990–1993	Invasive	338	39	261	Yes	Yes	Yes	Yes	Yes	Yes	Yes	No	NA	Partners/AFI
Ngelangel *et al* (1998), Philippines	1991–1993	Invasive	323	33	381	Yes	Yes	Yes	Yes	Yes	Yes	Yes	No	NA	AFIOC use
UK National Case–Control Study of Cervical Cancer (2003), UK	1984–1988	Invasive	391	180	923	Yes	Yes	No	No	Yes	Yes	Yes	No	Yes	All

OC=oral contraceptive (use); AFI=age at first intercourse; AFB=age at first birth; HPV=human papilloma virus (status); SP=sexual partners; Py=parity; Sm=smoking; SES=socio-economic status; Eth=ethnicity; Ctr=center; NA=not applicable.

* All studies adjusted for age and screening: as appropriate for each analysis

** All studies adjusted for age and screening: additionally adjusted for SES, parity, HPV for OC analysis.

# Additionally adjusted for induced abortion and marital status for adenocarcinoma+this pooled analysis includes data from 10 studies and all analyses were restricted to HPV positive women; two of these studies are included individually in meta-analyses where pooled data were not available (Chichareon, Ngelangel).

.

Figure 1 shows summary OR in relation to sexual behaviour, reproductive factors, oral contraceptive use and smoking status, based on data from between three and six studies. Both histological types of cervical cancer showed a strong association with the number of sexual partners, with cancer risk increasing with the increasing number of partners. Summary OR (and 95% CIs) for three or more lifetime partners compared with one partner were 1.94 (1.35–2.79) for adenocarcinoma and 2.44 (1.94–3.07) for squamous cell carcinoma. There were no significant differences between the results for adenocarcinoma and for squamous cell carcinoma. Early age at first intercourse was associated with increased risk of both types of cervical cancer, although the association was stronger for squamous cell carcinoma (OR for age at first intercourse of less than 17 years compared with more than 20 years 1.41 (0.99–2.00) for adenocarcinoma and 2.32 (1.89–2.85) for squamous cell carcinoma; the difference between these ORs was statistically significant (*P*=0.009)).

Parity was strongly related to the risk of squamous cell carcinoma (summary OR for three or more live births or full-term pregnancies compared with none 2.71 (2.08–3.53)). It was less strongly related to the risk of adenocarcinoma, although there was still a statistically significant association (OR for parity of three or more 1.51 (1.02–2.22)), and there appears to be a trend of increasing risk with increasing parity for adenocarcinoma as for squamous cell carcinoma. The difference between the OR for adenocarcinoma and for squamous cell carcinoma in relation to parity of three or more compared to none was statistically significant (*P*=0.006). Age at first birth was not clearly related to either squamous cell or adenocarcinoma of the cervix.

Duration of use of oral contraceptives was strongly related to risk for both adenocarcinoma and squamous cell carcinoma (OR for 10 or more years use compared with never use, 2.19 (1.58–3.02) and 2.02 (1.72–2.37), respectively), with no significant difference between the results for the two cancer types.

Compared to never smokers, the risk of squamous cell carcinoma was significantly increased in ever smokers (summary OR=1.23 (1.09–1.40) and in current smokers (summary OR=1.47 (1.15–1.88), although not in past smokers (summary OR=0.93 (0.69–1.25)). Adenocarcinoma risk was not associated with smoking status (summary OR=0.88 (0.69–1.11) for ever smokers, 0.82 (0.60–1.11) for current smokers and 0.88 (0.63–1.21) for past smokers compared to never smokers). There was a statistically significant difference between the risks for squamous cell and for adenocarcinoma for ever smoking (*P*=0.003) and for current smoking (*P*=0.001).

Statistically significant heterogeneity between studies was present in eight out of the 28 groups of studies (*P*-values for significant heterogeneity between studies: squamous cell carcinoma, >3 partners *P*=0.002, parity 1–2 *P*=0.03, age at first birth ⩽19 years *P*=<0.0001, <5 years oral contraceptive use *P*=0.03, past smoking *P*=0.04; adenocarcinoma, parity 1–2 *P*=<0.0001, age at first birth ⩽19years *P*=0.03, <5 years oral contraceptive use *P*=0.04).

The individual study OR for ever, past and current smokers compared to never smokers are shown in Figure 2. There was statistical heterogeneity of marginal significance between individual studies in one group only (squamous cell carcinoma in relation to past smoking; *P*=0.04).

Data on smoking intensity were available from two studies only: the summary risk of squamous cell carcinoma increased with increasing intensity of smoking (summary OR 1.22 (0.91–1.65) and 1.39 (1.01–1.91) for less than 20 and 20 or more cigarettes per day, respectively, compared to never smokers). The risk of adenocarcinoma was not significantly increased for either group of intensity of smoking compared to never smokers (summary OR 0.80 (0.56–1.13) and 0.77 (0.53–1.13) for less than 20 and 20 or more cigarettes per day, respectively.) There was a statistically significant difference between the results for squamous cell and for adenocarcinoma for both levels of intensity (less than 20 cigarettes per day, *P*=0.04; 20 or more cigarettes per day, *P*=0.01). No heterogeneity between studies was present in any group. Only three studies published results according to duration of smoking, and of these only one (Green *et al*, 2003) published results for duration of smoking restricted to current smokers. Because of the difference in risk seen for squamous cell cervical cancer between current and past smokers, it was not considered appropriate to combine the results for duration of smoking.

## DISCUSSION

The results of this meta-analysis show consistent qualitative differences between the risks for squamous cell and adenocarcinomas of the cervix in relation to cigarette smoking. Smoking appears to be a risk factor for squamous cell carcinoma, with an increased risk of around 1.5 for current smokers, but not for adenocarcinoma.

The other risk factors investigated did not differ qualitatively between squamous cell and adenocarcinomas; both types of cervical cancer were strongly related to the number of sexual partners and to duration of oral contraceptive use, and both were related to early age at first intercourse and to parity. Neither type of cervical cancer was related to age at first birth in this analysis. Together with strong evidence that HPV infection is a major, and probably a necessary, causal factor for both squamous cell and adenocarcinoma of the cervix, these findings confirm the impression from recent individual studies that the two main histological types of cervical cancer share the majority of risk factors. The risk factors for cervical adenocarcinoma differ substantially from those for endometrial adenocarcinoma; high parity and the use of oral contraceptives decrease the risk of endometrial cancer, and there is no evidence for an association between endometrial cancer and sexual behaviour or HPV infection (Altekruse *et al*, 2003; Green *et al*, 2003; Kjaer and Brinton, 1993).

This meta-analysis of published observational data has a number of limitations (Egger *et al*, 1998). The most serious is the difference between studies in adjustment for possible confounding factors and for HPV exposure or infection (see Table 1). The four studies included in the smoking meta-analysis, however, all gave results restricted to HPV-positive women (Plummer *et al*, 2003) or adjusted for HPV status (Lacey *et al*, 2001) or for lifetime number of sexual partners, a reasonable surrogate for HPV exposure (Brinton *et al*, 1993; Green *et al*, 2003). Two studies did not provide results adjusted for HPV infection or exposure (WHO, 1985; Brinton *et al*, 1986; Thomas and Ray, 1996); both were included only in the meta-analysis of oral contraceptive use, and the results of this analysis were not materially altered when these two studies were omitted. Differences in the risk factor categories used, for example for duration of oral contraceptive use, may also contribute to the statistical heterogeneity seen between studies in some groups. Overall, the number of studies that have published results in a similar way for both squamous cell and adenocarcinoma of the cervix is small, and for some of the analyses the number of studies was very limited. This meant that it was not feasible to investigate heterogeneity between studies formally with respect to different study characteristics. For all of these reasons, the magnitude of the summary odds ratios should be interpreted cautiously.

Observed differences between the risks for squamous cell and for adenocarcinomas could be due to selection or reporting biases, or to differential residual confounding with other risk factors. Cervical screening, for example, is thought to be more effective in detecting squamous cell than adenocarcinomas (Mitchell *et al*, 1995; Bergstrom *et al*, 1999); while all studies in this meta-analysis provided results adjusted for screening, the extent of adjustment was variable. However, factors such as these seem unlikely to explain the differences observed in relation to smoking as the two histological types did not differ substantially in the analyses for sexual behaviour, oral contraceptive use or reproductive factors. Some of these factors, such as oral contraceptive use, are known to be related to cervical screening (Eaker *et al*, 2001). The similarities with respect to other risk factors also suggest that there is unlikely to have been substantial misclassification of cervical adenocarcinomas in these studies (Green *et al*, 2003).

Some other epithelial cancers, for example those of the nasal cavity, the oesophagus and possibly the lung, appear to show differences between squamous cell and adenocarcinomas in relation to smoking, with the effect of smoking being greater for squamous cell tumours (IARC, 2004, in press). The results of this meta-analysis of available published data suggest that smoking increases the risk of squamous cell carcinoma of the cervix, but has no clear effect on the risk of adenocarcinoma of the cervix. Further studies are needed to confirm this finding.

## References

[bib1] Altekruse SF, Lacey JVJ, Brinton LA, Gravitt PE, Silverberg SG, Barnes WAJ, Greenberg MD, Hadjimichael OC, McGowan L, Mortel R, Schwartz PE, Hildesheim A (2003) Comparison of human papillomavirus genotypes, sexual, and reproductive risk factors of cervical adenocarcinoma and squamous cell carcinoma: Northeastern United States. Am J Obstet Gynecol 188: 657–663 12634637 10.1067/mob.2003.132

[bib2] Bergstrom R, Sparen P, Adami HO (1999) Trends in cancer of the cervix uteri in Sweden following cytological screening. Br J Cancer 81: 159–166 10487628 10.1038/sj.bjc.6690666 PMC2374360

[bib3] Berrington A, Cox DR (2003) Generalised least squares for the synthesis of correlated information. Biostatistics 4: 423–431 12925509 10.1093/biostatistics/4.3.423

[bib4] Brinton LA, Herrero R, Reeves WC, de Britton RC, Gaitan E, Tenorio F (1993) Risk factors for cervical cancer by histology. Gynecol Oncol 51: 301–306 8112636 10.1006/gyno.1993.1294

[bib5] Brinton LA, Huggins GR, Lehman HF, Mallin K, Savitz DA, Trapido E, Rosenthal J, Hoover R (1986) Long-term use of oral contraceptives and risk of invasive cervical cancer. Int J Cancer 38: 339–344 3744592 10.1002/ijc.2910380307

[bib6] Brinton LA, Reeves WC, Brenes MM, Herrero R, de Britton RC, Gaitan E, Tenorio F, Garcia M, Rawls WE (1990) Oral contraceptive use and risk of invasive cervical cancer. Int J Epidemiol 19: 4–11 2351522 10.1093/ije/19.1.4

[bib7] Chichareon S, Herrero R, Munoz N, Bosch FX, Jacobs MV, Deacon J, Santamaria M, Chongsuvivatwong V, Meijer CJ, Walboomers JM (1998) Risk factors for cervical cancer in Thailand: a case–control study. J Natl Cancer Inst 90: 50–57 9428783 10.1093/jnci/90.1.50

[bib8] Clifford GM, Smith JS, Plummer M, Munoz N, Franceschi S (2003) Human papillomavirus types in invasive cervical cancer worldwide: a meta-analysis. Br J Cancer 88: 63–73 12556961 10.1038/sj.bjc.6600688 PMC2376782

[bib9] Cox DR, Snell EJ (1989) Analysis of Binary Data. London: Chapman & Hall

[bib10] Eaker S, Adami HO, Sparen P (2001) Reasons women do not attend screening for cervical cancer: a population-based study in Sweden. Prev Med 32: 482–491 11394952 10.1006/pmed.2001.0844

[bib11] Egger M, Schneider M, Davey SG (1998) Spurious precision? Meta-analysis of observational studies. BMJ 316: 140–144 9462324 10.1136/bmj.316.7125.140 PMC2665367

[bib12] Green J, Berrington de Gonzalez A, Sweetland S, Beral V, Chilvers C, Crossley B, Deacon J, Hermon C, Jha PK, Mant D, Peto J, Pike MC, Vessey M (2003) Risk factors for adenocarcinoma and squamous cell carcinoma of the cervix in women aged 20–44 years: the UK National Case–Control Study of Cervical Cancer. Br J Cancer 89: 2078–2086 14647141 10.1038/sj.bjc.6601296 PMC2376844

[bib13] IARC Working Group on the Evaluation of Carcinogenic Risks to Humans 1999 Hormonal Contraception and Post-Menopausal Hormonal Therapy. IARC Monographs on the Evaluation of Carcinogenic Risks to Humans, Vol. 72 Lyon: International Agency for Research on Cancer

[bib14] IARC 2004 Tobacco Smoke and Involuntary Smoking. IARC Monographs on the Evaluation of Carcinogenic Risks to Humans, Vol. 83 Lyon: International Agency for Research on Cancer (in press) PMC4781536 15285078

[bib15] Kjaer SK, Brinton LA (1993) Adenocarcinomas of the uterine cervix: the epidemiology of an increasing problem. Epidemiol Rev 15: 486–498 8174668 10.1093/oxfordjournals.epirev.a036131

[bib16] Lacey JV, Brinton L, Abbas FM, Barnes WA, Gravitt PE, Greenberg MD, Greene SM, Hadjimichael O, McGowan L, Mortel R, Schwartz PE, Silverberg SG, Hildesheim A (1999) Oral contraceptives as risk factors for cervical adenocarcinomas and squamous cell carcinomas. Cancer Epidemiol Biomarkers Prev 8: 1079–1985 10613340

[bib17] Lacey JVJ, Frisch M, Brinton LA, Abbas FM, Barnes WA, Gravitt PE, Greenberg MD, Greene SM, Hadjimichael OC, McGowan L, Mortel R, Schwartz PE, Zaino RJ, Hildesheim A (2001) Associations between smoking and adenocarcinomas and squamous cell carcinomas of the uterine cervix (United States). Cancer Causes Control 12: 153–161 11246844 10.1023/a:1008918310055

[bib18] Mitchell H, Medley G, Gordon I, Giles G (1995) Cervical cytology reported as negative and risk of adenocarcinoma of the cervix: no strong evidence of benefit. Br J Cancer 71: 894–897 7710961 10.1038/bjc.1995.172 PMC2033741

[bib19] Munoz N, Franceschi S, Bosetti C, Moreno V, Smith JS, Shah KV, Meijer CJ, Bosch F (2002) Role of parity and human papillomavirus in cervical cancer: the IARC multicentric case–control study. Lancet 359: 1093–1101 11943256 10.1016/S0140-6736(02)08151-5

[bib20] Ngelangel C, Munoz N, Bosch FX, Limson GM, Festin MR, Deacon J, Jacobs MV, Santamaria M, Meijer CJ, Walboomers JM (1998) Causes of cervical cancer in the Philippines: a case–control study. J Natl Cancer Inst 90: 43–49 9428782 10.1093/jnci/90.1.43

[bib21] Parazzini F, La Vecchia C (1990) Epidemiology of adenocarcinoma of the cervix. Gynecol Oncol 39: 40–46 2227571 10.1016/0090-8258(90)90396-3

[bib22] Plummer M, Herrero R, Franceschi S, Meijer CJ, Snijders PJ, Bosch FX, de Sanjose S, Munoz N (2003) Smoking and cervical cancer: pooled analysis of the IARC multi-centric case–control study. Cancer Causes Control 14: 805–814 14682438 10.1023/b:caco.0000003811.98261.3e

[bib23] Sasieni P, Adams J (2001) Changing rates of adenocarcinoma and adenosquamous carcinoma of the cervix in England. Lancet 357: 1490–1493 11377601 10.1016/S0140-6736(00)04646-8

[bib25] Thomas DB, Ray RM (1996) Oral contraceptives and invasive adenocarcinomas and adenosquamous carcinomas of the uterine cervix. The World Health Organization Collaborative Study of Neoplasia and Steroid Contraceptives. Am J Epidemiol 144: 281–289 8686697 10.1093/oxfordjournals.aje.a008923

[bib26] Walboomers JM, Jacobs MV, Manos M, Bosch F, Kummer A, Shah K, Snijders PJ, Peto J, Meijer CJ, Munoz N (1999) Human papillomavirus is a necessary cause of invasive cervical cancer worldwide. J Pathol 189: 12–19 10451482 10.1002/(SICI)1096-9896(199909)189:1<12::AID-PATH431>3.0.CO;2-F

[bib27] WHO (1985) Invasive cervical cancer and combined oral contraceptives. WHO collaborative study of neoplasia and steroid contraceptives. Br Med J Clin Res Ed 290: 961–965 10.1136/bmj.290.6473.961 PMC1418262 3919869

